# Scoring sleep using respiration and movement-based features

**DOI:** 10.1016/j.mex.2022.101682

**Published:** 2022-03-31

**Authors:** H. Kloefkorn, L.M. Aiani, S. Hochman, N.P. Pedersen

**Affiliations:** a Department of Physiology, School of Medicine, Emory University, 615 Michael Street, Atlanta, GA 30033, United States; b Department of Neurology, School of Medicine, Emory University, Atlanta, GA, United States

**Keywords:** Sleep scoring, Noninvasive, Rodent, Electric field sensor

## Abstract

Rules derived from standard Rechtschaffen and Kales criteria were developed to accurately score rodent sleep into wake, rapid eye movement (REM) sleep, and non-REM sleep using movements detected by non-contact electric field (EF) sensors.

• Using this method, rodent sleep can be scored using only respiratory and gross body movements as a validated, non-invasive alternative to electrode techniques.

• The methodology and rules established for EF sensor-based sleep scoring were easily learned and implemented.

• Examples of expert-scored files are included here to help novice scorers self-train to score sleep.

Though validated in mice, sleep scoring using respiratory movements has the potential for application in other species and through other movement-based technologies beyond EF sensors.

## Graphical abstract

**Figure:**
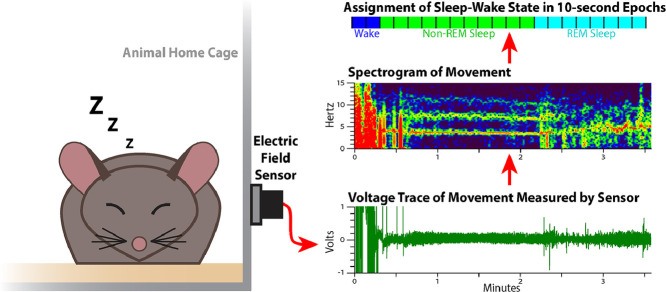

Specifications table

**Table:** 

Subject Area:	Neuroscience
More specific subject area:	Sleep
Method name:	Sleep Scoring Using Respiration- and Movement-Based Features
Name and reference of original method:	Kloefkorn H, Aiani L, Lakhani A, et al. Noninvasive Three-State Sleep-Wake Staging in Mice using electric field sensors. *J Neurosci Methods*. 2020;344. doi:https://doi.org/10.1016/j.jneumeth.2020.108834
Resource availability:	Supplementary reference files of expert-score data can be used to help novice scorers self-train to score sleep.

## Method details

There are unique movement patterns for each arousal state that can be measured using several technologies [1], [2], [3], [4]. The sleep scoring method presented here uses movement-based detection of respiratory and gross body movements to distinguish between wake, rapid eye movement (REM) sleep, and non-REM sleep in mice without electroencephalogram (EEG), electromyogram (EMG) or video. Here, we describe sleep data collected using noncontact electric field (EF) sensors placed outside mouse home cages. The EF sensors translate movement-related disturbance in the local electric field into voltage traces that can be analyzed using time- and frequency-based approaches. Movement during the wake state is determined exclusively using the overt voltage signature of gross body movement, but the movement-based differences between REM and non-REM sleep are more subtle incorporating features from both respiration and gross body movement. Described here are the rules for scoring sleep into three stages of wake, non-REM and REM sleep using movement- and respiration-based data collected by EF sensors.

### Summary of validation for electric field (EF) sensors to score sleep

In another study [4], EF sensors were validated against EEG and EMG to accurately quantify sleep with expert scorer agreement above 93%. Briefly, mice were implanted with EEG and EMG electrodes and single-housed in barrel-style acrylic cages. EF sensors were affixed to the exterior surface of the chamber to continuously translate animal movement into a voltage trace synced with EEG/EMG. From these synchronized data, criteria were created by expert scorers for scoring sleep according to Rechtschaffen and Kales [5]. Novices were able to score sleep from EF sensor-collected movement data with high agreement (> 87.6%) to expert scorers using only the reference training material provided (Supplemental Files 1, 2, 3, 4, and 5) and instructions described below. Once validated, the EF sensors were also placed on standard vivarium home cages of completely naïve mice as proof they could accurately quantify sleep without EEG/EMG in a traditional home cage setting.

### EF sensor recordings of wake, REM sleep, and non-REM sleep exhibit unique patterns

**Fig. 1:**
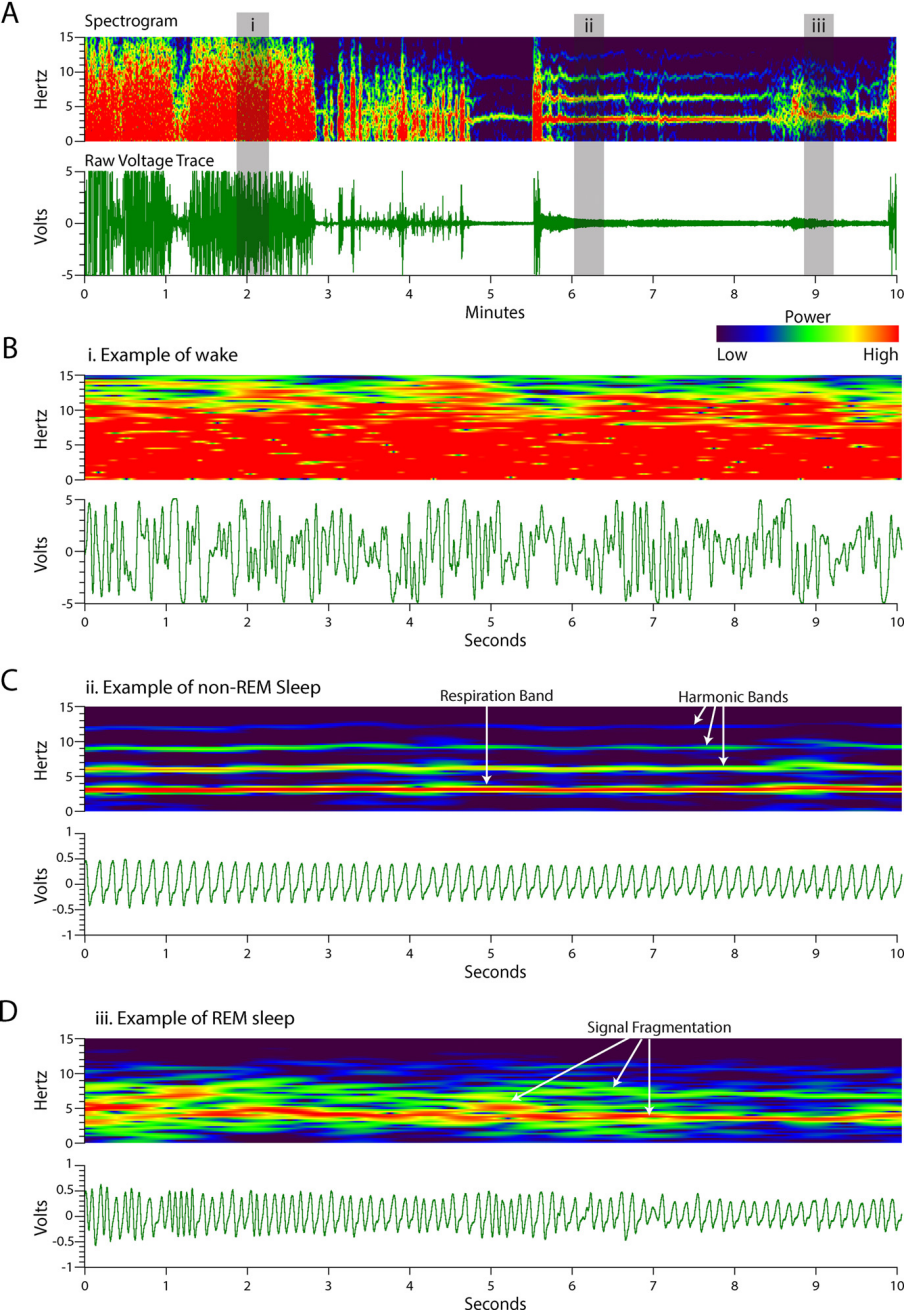
**Spectrogram and voltage traces of wake, Non-REM sleep, and REM sleep.** Data from the EF sensors can be visualized as a voltage trace or spectrogram. Spectrograms are created through fast Fourier transformation of the voltage trace and contains information about the relative power (color intensity) for the frequencies (y-axis) that exist in the voltage trace across time (x-axis). (A) The spectrogram has been created from the voltage trace and encompasses wake, non-REM sleep, and REM sleep states, examples of which are called out by the gray box. (B) Magnified view of the (i) grey box from panel A. Likewise (C) and (D) are (ii) and (iii) and show non-REM and REM sleep, respectively. Note difference in scale of voltage trace panels in C and D compared to A and B. In Panel C, the red frequency band at ∼3.5 Hz corresponds with respiratory rate. Spectrograms of respiration typically include less powerful harmonic bands at multiples of the primary signal frequency (white arrows).

Scoring sleep is performed using the raw voltage trace collected by the EF sensors and a spectrogram created from the voltage trace. A spectrogram displays the frequency power information of a signal as a colormap indicating the relative power of a given frequency (y-axis) at a given time (x-axis) (see Fig. 1). Spectral power is calculated using the fast Fourier transform (FFT) of sliding windows along the time axis (x-axis).

Fig. 1 provides an example of data displayed as raw voltage traces and corresponding spectrograms that includes epochs of wake, non-REM and REM sleep. The wake state encompasses mouse movements with highly variable magnitude and frequency (Fig. 1B). Common movements include eating, drinking, grooming, locomotion, sniffing, and rearing and range from very low frequencies (< 1 Hz) through the highest typical movement frequency (> 10 Hz). Overall the recorded voltage trace exhibits patterns with relatively large amplitude and erratic shape with high variability in amplitude and width of each peak. The corresponding spectrograms depict large areas of high-powered signal solidly covered frequencies from near 0 Hz to above 15 Hz.

Using respiratory and other movement-based features, sleep is differentiated from wake initially by the absence of larger movements associated with wake. Thus, smaller movements, such as respiration and small twitches, can be captured during sleep and used to delineate REM from non-REM sleep (Fig. 1D and C, respectively).

**Fig. 2:**
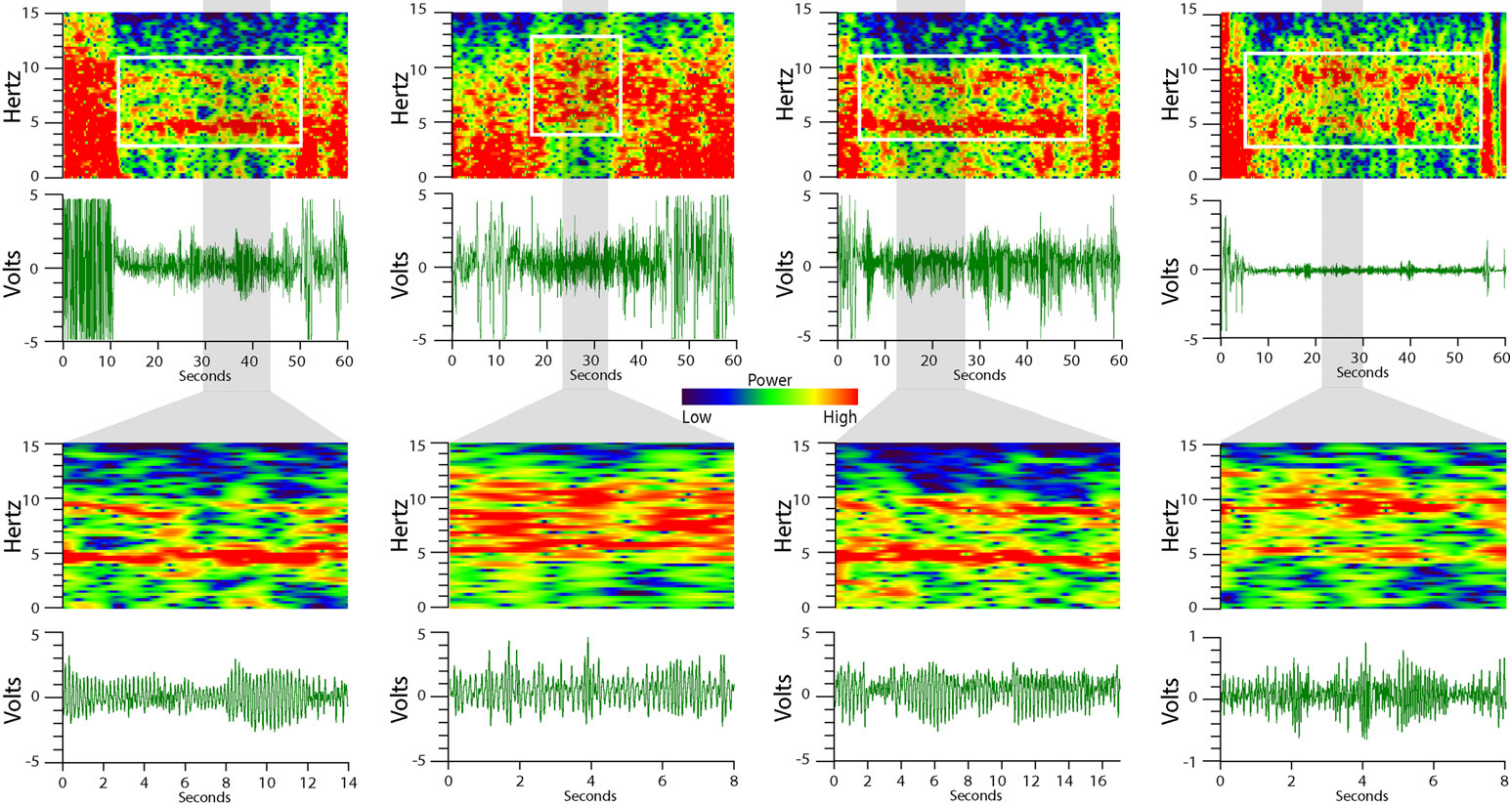
**Non-REM sleep respiration voltage traces vary.** While respiratory voltage waveforms vary with animal orientation relative to EF sensor placement, frequency accuracy is not dependent on the voltage trace shape.

Non-REM sleep is characterized by the cyclic voltage trace the EF sensors generate reflecting respiration (Fig. 1C). The shape of each peak in the voltage trace during non-REM sleep is consistent within a sleeping event, but can change across sleeping events as an animal resituates (Fig. 2). The average non-REM sleep voltage amplitude is often an order of magnitude lower than seen during wake behaviors. The unifying feature of non-REM sleep is the local consistency of signal shape, amplitude, and frequency associated with respiration rate. On the spectrogram, this appears as a narrow band of high-power frequency centered around 2–4 Hz for mice, sometimes with harmonic bands (white arrows) appearing at multiples of the respiration rate (Fig. 1C).

**Fig. 3:**
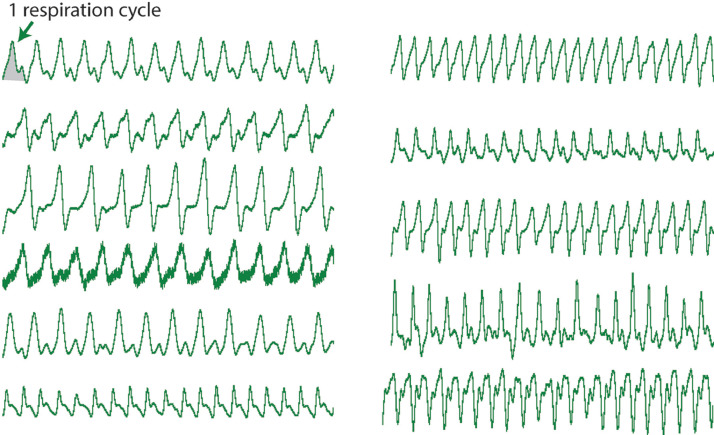
**Wake spectrograms and voltage traces.** The left column images are over longer timescales than the right column images. Wake voltage trace patterns are highly erratic in both amplitude and frequency, reflective of complex gross body movement. Likewise, the wake spectrogram has more power (i.e. more red) in the 0–15 Hz range than in other behavior states.

**Fig. 4:**
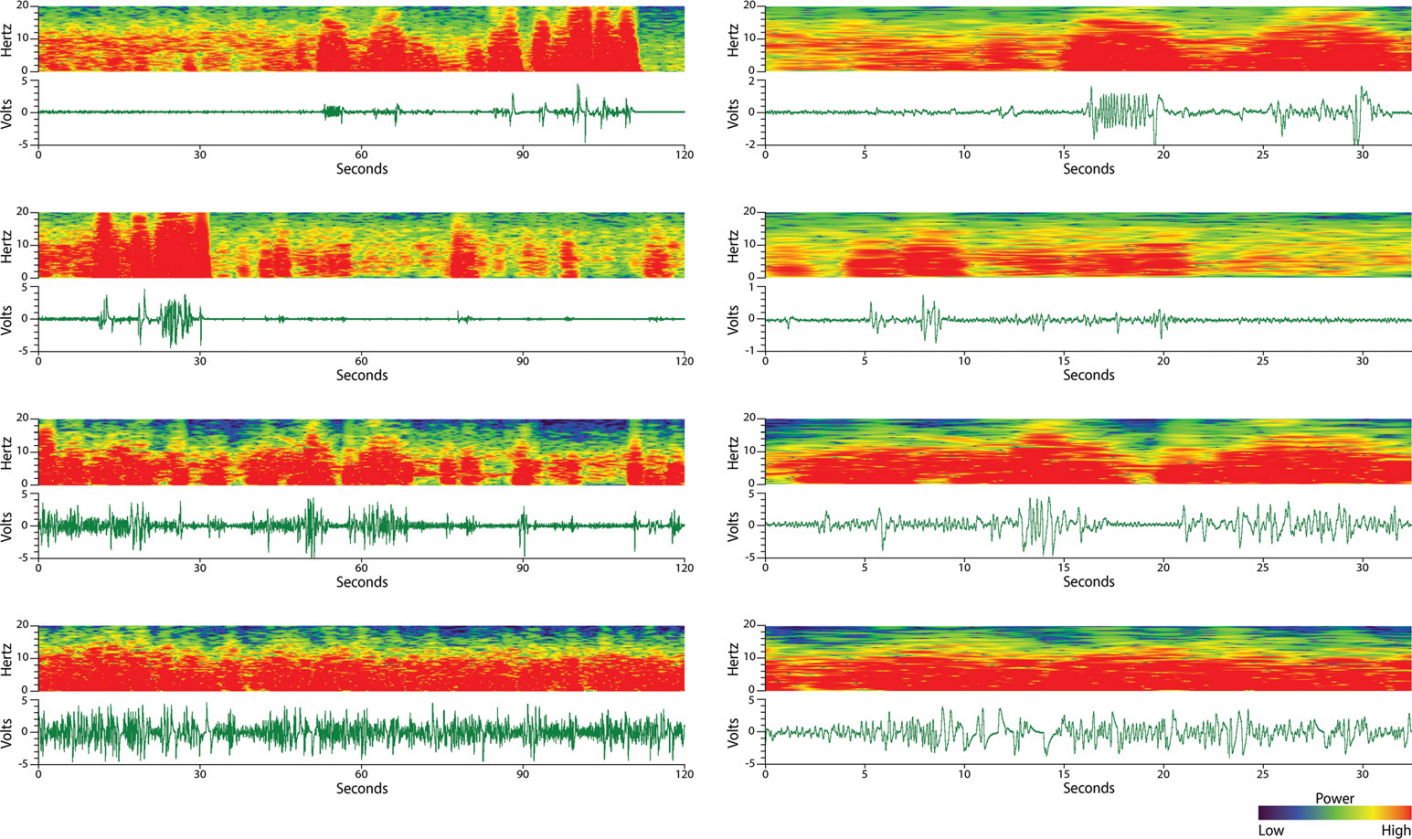
**Non-REM sleep and REM sleep spectrograms and voltage traces.** In both panels, the left column of images encompasses longer timescales than in the right column of images. (A) Non-REM sleep voltage traces are consistent and cyclic, reflecting respiration. Non-REM sleep spectrograms exhibit a strong (i.e. more red) frequency band at respiration rate, sometimes with harmonic bands at mathematical multiples. (B) REM sleep voltage traces are more varied in amplitude and frequency than non-REM, but less than wake. During REM sleep, twitches (red arrow) can be discerned in the voltage trace. REM sleep spectrogram patterns are typically spotty or fragmented with the bulk of the signals between the respiration rate and 10 Hz. Rarely, REM sleep signals will appear below respiration rate and are largely attributed to large twitches.

REM sleep is differentiated from non-REM sleep by an increase in the variability of respiration (tonic REM sleep) and the addition of muscular twitches (phasic REM sleep) [6], [7], [8], [9]. The commonality of all REM sleep patterns observed using the EF sensors is a slight increase in the signal frequency and amplitude variability relative to the immediately preceding non-REM sleep (Fig. 1D). This appears on the spectrogram as a fragmented or spotty pattern of frequency power, less powerful than wake, spanning frequencies ranging from that of respiration (typically 2–4 Hz) through nearly 10 Hz (Fig. 1D). Aside from local amplitude differences, REM sleep is further differentiated from wake behaviors by an absence of very low-frequencies (< 1 Hz) on the spectrogram; where wake would have powerful frequency representation to nearly 0 Hz, REM sleep spectrograms rarely have any moderately represented frequencies below the respiration rate, with large twitches being the exception (see red boxes in Figs. 4B and 7A). More examples of wake, non-REM sleep, and REM sleep can be found in Figs. 3 and 4.

**Fig. 5:**
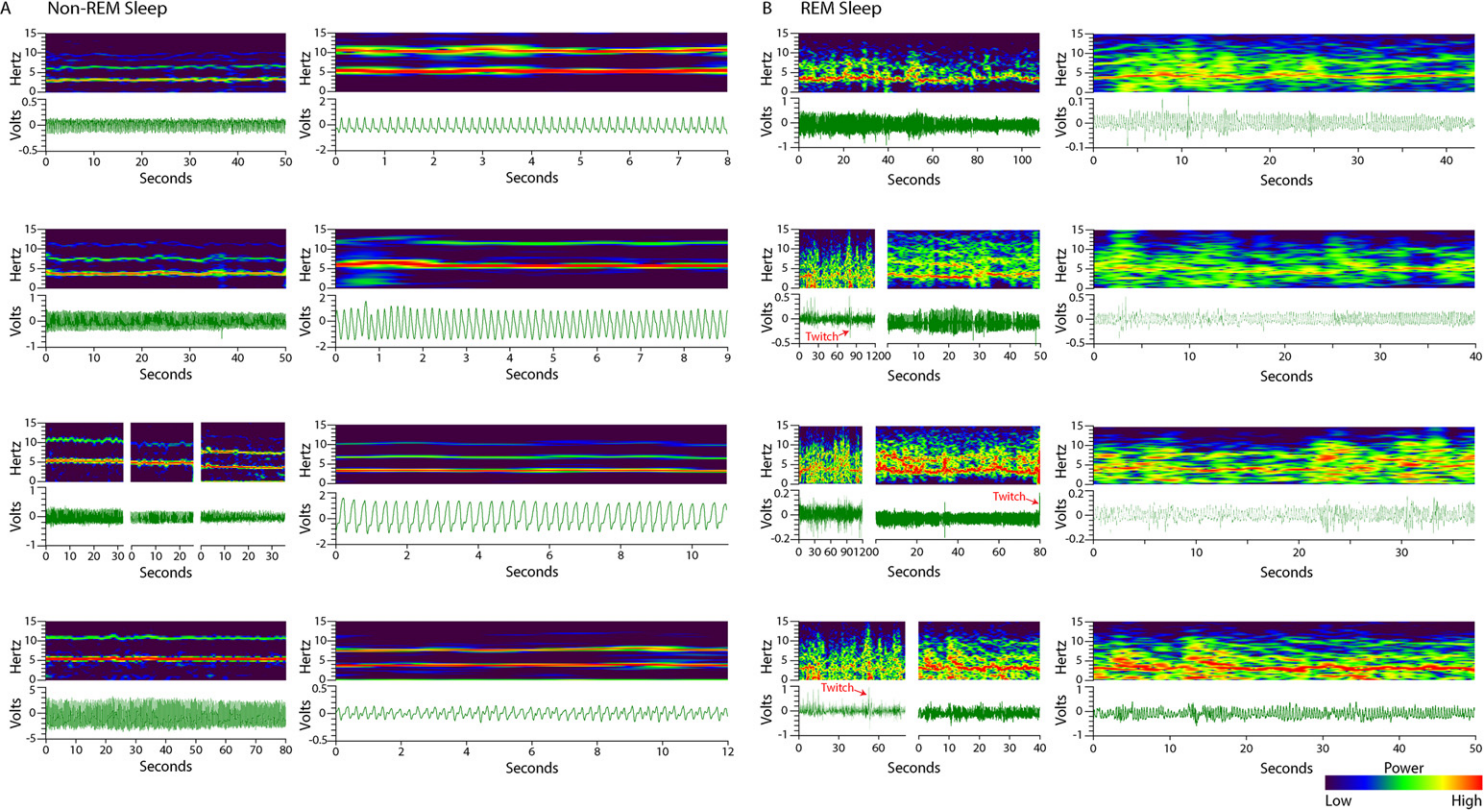
**Grooming behavior.** Grooming behavior (white boxes in the top row) often has strong rhythmic components which can be mistakenly scored as non-REM sleep by novice sleep scorers. Grooming is distinct from non-REM sleep because the voltage trace amplitude as well as the resulting spectrogram frequency band are larger and more erratic then during non-REM sleep. Furthermore, grooming behavior is commonly sandwiched between other large motor movements and rarely lasts more than 1 min before the animal pauses or changes behavior. Non-REM sleep is a less powerful, more consistent behavior that typically lasts more than 1 min before interruption.

Grooming, a characteristic rhythmic motor behavior observed during wake, can be mistakenly identified as non-REM sleep by novice scorers. Grooming specifically has strong rhythmic components and will sometimes appear on the spectrogram as a fragmented, wide band centered between 5 and 10 Hz (Fig. 5). Though these grooming patterns may share similarities with non-REM sleep voltage traces, the frequency pattern and context of grooming is distinct: the voltage trace amplitude and spectrogram frequencies are higher, more powerful, and more erratic than during non-REM sleep. Furthermore, grooming rarely lasts more than one minute before the animal pauses or continues performing other waking behavior. In contrast, an isolated non-REM event lasting less than one minute surrounded by large body movements is highly unusual.

Since the EF sensor voltage trace patterns are unique for each arousal state, it may be possible for experts to score sleep using only the voltage trace, as can be done with sleep scored using whole body plethysmography [1]. However, we did not collect any data regarding the accuracy of scoring sleep using the EF sensor voltage trace alone.

### Sleep scoring criteria

The rules for scoring sleep using EF sensors were developed during the validation study using EEG/EMG [4]. Briefly, synchronous data was collected for EEG/EMG and EF sensors for four mice. EEG/EMG data were sleep scored for two animals (12 h per animal) according to Rechtschaffen and Kales criteria [5] in which scoring was performed by dividing the data into equal epochs of time (10 s epochs) and assigning an arousal state to each epoch. Based on the EEG/EMG sleep scores, experts in sleep analysis defined four rules for the matching EF data for each arousal state:

1. Each epoch is scored based on the majority of the state present in that epoch using the voltage trace and spectrogram guidance stated above. If a 10 s epoch consists of 7 s of wake and 3 s of non-REM, it will be scored as wake. If an epoch is evenly divided between two states, it will be scored as the immediately previous state (Fig. 6).

**Fig. 6:**
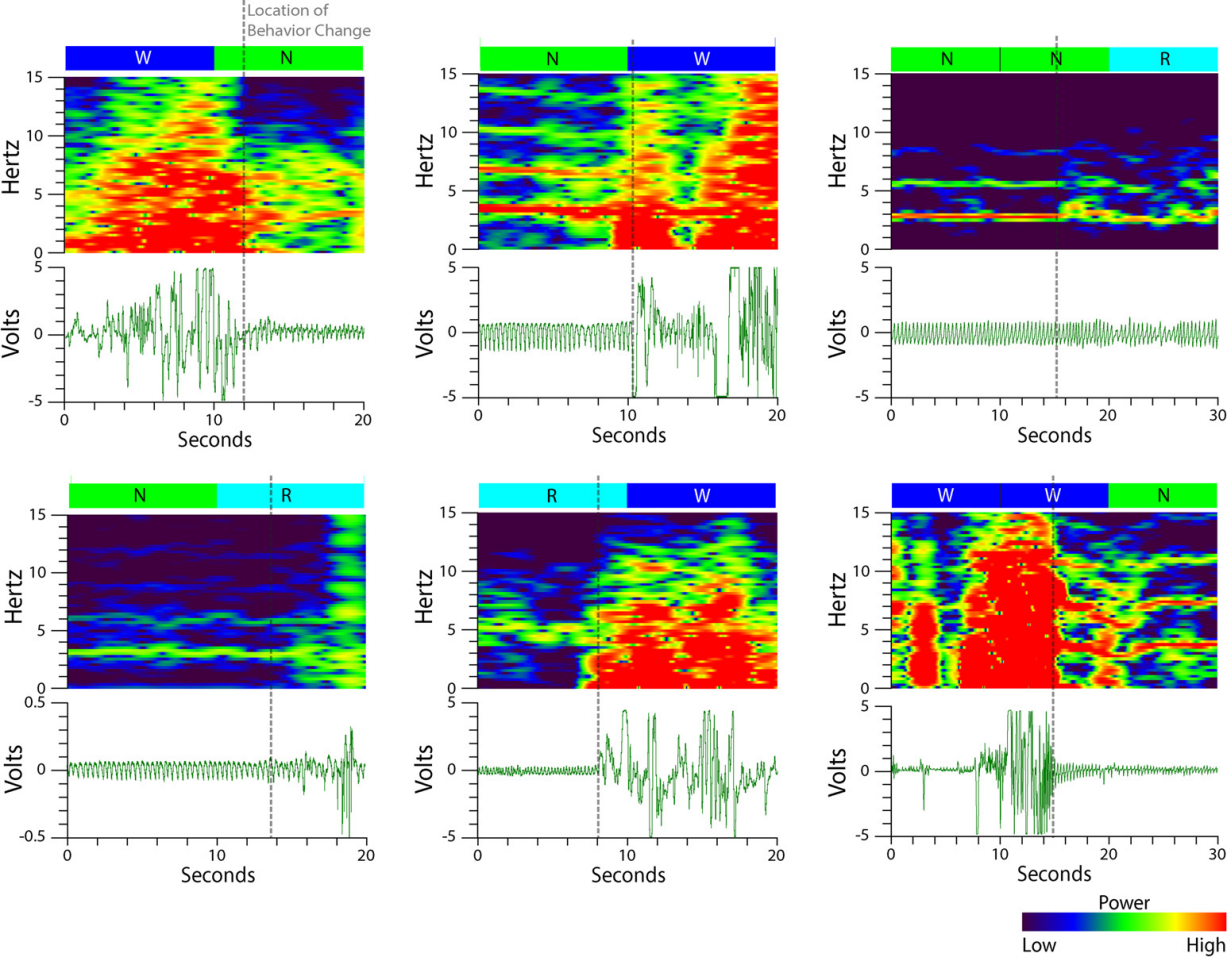
**Scoring the majority of an epoch.** Sleep is quantified by dividing up the data into epochs of time (in this case, 10 s epochs) and assigning an arousal state to that epoch. The epoch is scored according to what behavior occupies its temporal majority. The boxes above the spectrograms represent the 10 s epochs, falling along the x-axis (time) as they were predetermined from the beginning of the data which rarely fall cleanly at a behavior change. *W* = wake (blue), *N* = non-REM sleep (green), and *R* = REM sleep (cyan). The grey dotted lines are approximately where a behavior changed according to the voltage trace. The voltage trace is used to define the time at which there was a change in behavior because the spectrogram is temporally less precise due to the temporal leak and thus bleeding of spectra between epoch caused by the moving FFT window. If the change in behavior happens exactly halfway through a 10 s epoch (right column of images), that epoch is scored as the previous behavior and the score changes at the next epoch.
2. REM sleep cannot transition directly from wake. This rule was developed for normal sleep behavior and may not accurately reflect arousal changes in mice with narcolepsy or other sleep abnormalities. However, as EF sensors can detect unusual sleep episodes through movement, this rule can be adapted to fit several models.
3. Non-REM cannot be scored as a single epoch. Non-REM sleep is unique and must occur in two or more consecutive epochs to be defined as a state change from wake or REM sleep. Wake and REM sleep states are allowed to exist as single epochs as long as rules 1 and 2 are followed.
4. Single epochs of wake (brief arousals) are scored only if the wake-related movement represents the majority of the epoch (consistent with rule 1) and lasts for 10 s or more. Single epochs of wake (brief arousals) present a unique challenge to respiration- and movement-based sleep scoring criteria (Fig. 7). This special case is made because movement, especially a very short duration movement such as a twitch, does not always denote cortical arousal to wake (Fig. 7A). As confirmed by EEG during the validation study [4], a movement that lasted at least 10 s indicated cortical arousal. Movement during a non-REM sleep event that is close but does not solidly span 10 or more seconds (Fig. 7B), or instead being broken into a series of short (< 1 s) twitches clumped closely together (Fig. 7C), are not scored as a brief arousal. Clusters of brief movements or twitches can be differentiated from a continuous brief arousal by the respiration pattern between the twitches (blue arrows in Fig. 7C): brief movement clusters will have consistent respiration, similar to the surrounding non-REM pattern, between the twitch events while a continuous brief arousal will have no respiration patterns or highly variable respiration patterns between the peaks (Fig. 7D). While the most accurate scoring occurs when the voltage trace and spectrogram are consulted together, durations of brief arousals are best analyzed using the voltage trace because the spectrogram artificially widens movement events in the x-axis (time) due to the sliding window used to calculate the power spectrum. This phenomenon is illustrated in Fig. 7A when comparing the duration of twitches on the voltage trace versus the spectrogram

**Fig. 7:**
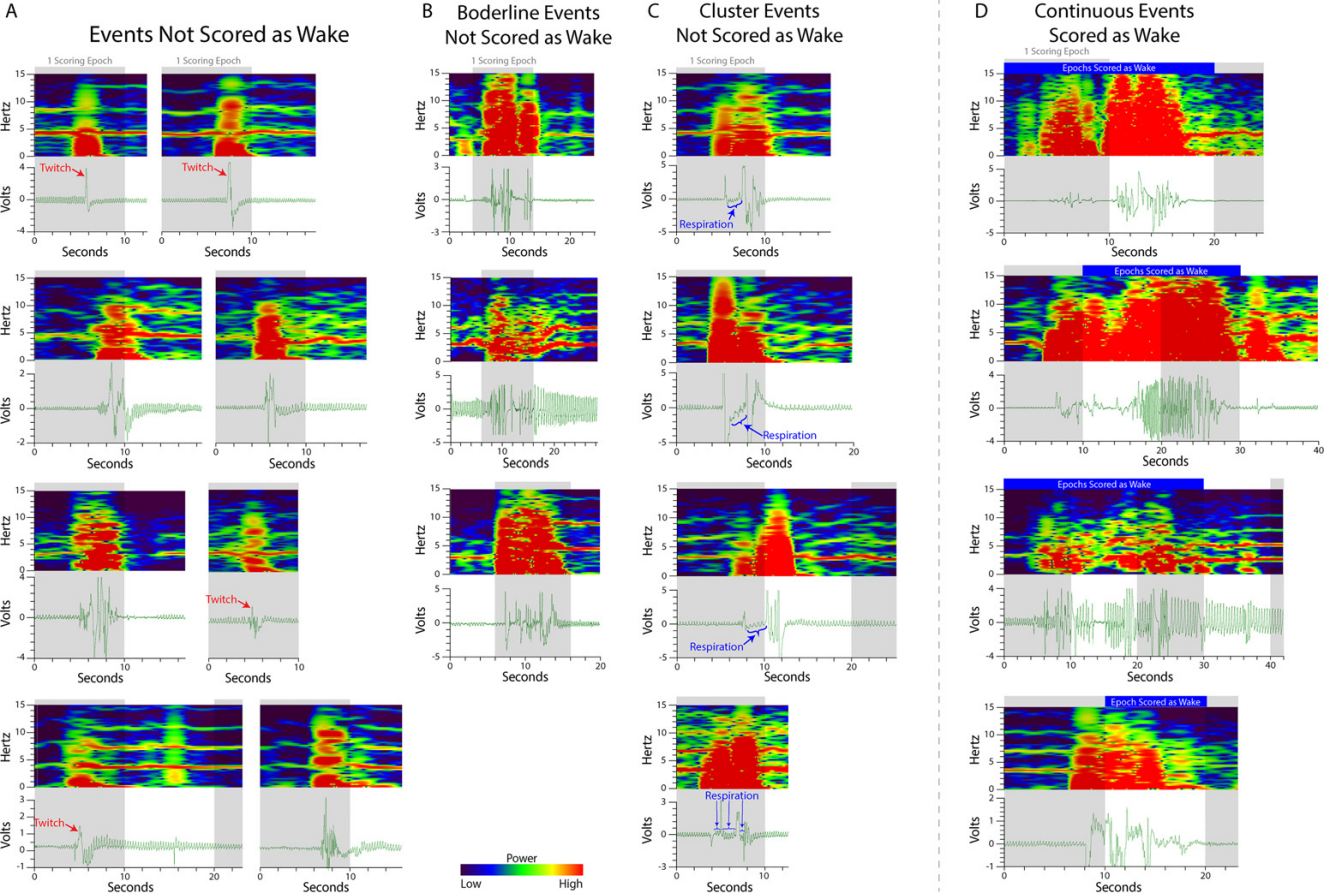
**Brief arousals do not always indicate cortical arousal.** Unlike EEG/EMG, brief movement arousals are more difficult to interpret using respiration and movement-based methods of sleep scoring. Validated by EEG/EMG, special scoring criteria were developed to best delineate movement-based brief movement arousals that likely pair with cortical arousals from those that do not. During scoring development, it was observed that EF sensors were especially sensitive to body twitches that show up with more spectral power (i.e. more red). Brief movement arousals less than 10 s in duration were unlikely to denote cortical arousal (A, B, and C). (A) Very short movement events rarely denote a cortical arousal and reflect common twitches (red arrows) during sleep. (B) Some brief movement arousal durations are slightly under the 10 s threshold and are difficult to score consistently, however they make up a small percentage of total events. (C) Clusters of very short (< 1 s) movements can be mistakenly interpreted as a single cumulative event breaking the 10 s rule. These cluster events are differentiated from continuous events (D) by the cyclic respiration (blue boxes) voltage trace between the twitches – indicating separate movement events. The grey boxes indicate 10 s epochs.

### Sleep scoring instructions using Spike2

As mentioned above, sleep scoring consists of assigning an arousal state for each epoch of time. This can be achieved through multiple different types of software capable of dealing with time series data and able to divide the data into epochs and graph it as both a voltage trace and a spectrogram. Here, we describe a way to score sleep using the software Spike2 (Version 8, Cambridge Electronic Design, Cambridge, UK) on a personal computer.

**Fig. 8:**
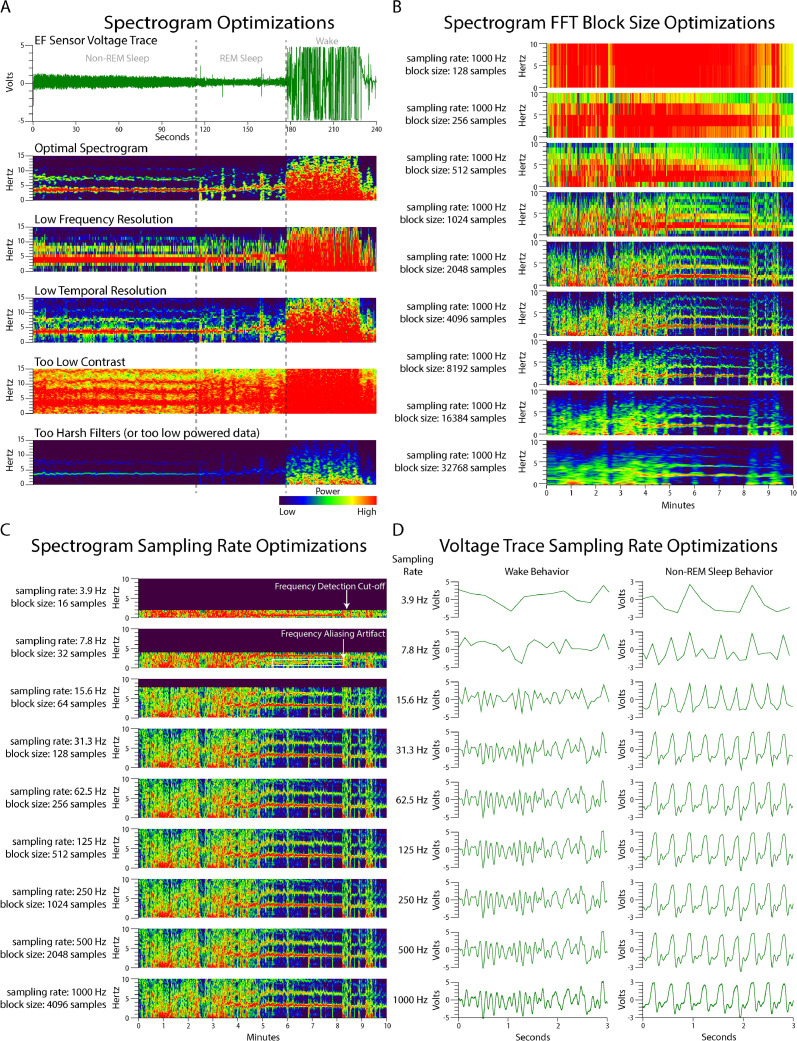
**Optimizing spectrogram to visualize respiration and movement data.**(A) The example raw voltage trace encompasses non-REM, REM, and wake states to best show how optimizing a spectrogram creates the clearest visual for each state. The optimal spectrogram was created using an FFT Hanning filter, displayed using a rainbow colormap, and optimized in Spike 2 with a block size of 4096, a pixel size of 1, a top dB of 60 (of options between 1 and 96), and range dB of 40 (of options between 1 and 96) for data collected at 1000 Hz. Poorly optimized spectrograms make accurate sleep scoring difficult or impossible. (B) This series of spectrograms all represent data collected at 1000 Hz with varying FFT block sizes. Block sizes of 2–4x the sampling frequency provide optimal visualization of frequency data. (C) This series of spectrograms represent down-sampled versions of the original 1000 Hz data with appropriate block sizes for each down-sampled rate. To distinguish between wake, non-REM, and REM sleep using frequency features alone, data should be sampled above 30 Hz. Below this rate, the FFT is unable to detect frequencies above ½ the sampling rate and cuts off (white arrow) the data. Likewise, a sampling rate less than 15 Hz creates an artificial harmonic at 1.5 Hz (white box) through aliasing errors, at least in the absence of an appropriate anti-aliasing filter. D) These voltage traces represent down-sampled versions of the original 1000 Hz data. Just as with the spectrogram, sampling rates less than 30 Hz are may be unable to capture critical data for scoring sleep.

Spike2 can both collect and analyze voltage traces captured by EF sensors [4] and import many file types. Once the voltage trace data is opened or imported into Spike2, the voltage trace channel should be duplicated. This duplicate channel can be converted into a spectrogram using the “Channel Draw Mode” function for simultaneous viewing of the voltage trace and spectrogram as shown in Fig. 1A. The ideal spectrogram settings will visually contrast wake, non-REM sleep, and REM sleep (Fig. 8A). Using the “Channel Draw Mode” menu, the desired channel can be selected and converted into a spectrogram (called a sonogram in Spike2) that can be optimized to best visualize relevant signal frequencies by changing several variables: the filter type, top dB, range dB, pixel window, and sliding window block size.

Spectrogram features are described in detail in the Spike2 instruction manual available from CED (http://ced.co.uk). Briefly, the “top dB” variable is a power ratio displayed in decibels (dB) which scales the colormap to increase power (lower number) or decrease power (higher number). The “range dB” value is a dB ratio representing the range of signal power within the data such that a lower number will alter the colormap to better delineation between frequencies close together. “Pixel Window” sets the pixel resolution of the spectrogram x-axis (time) and “Block Size” sets the number of data points used by the FFT along the y-axis (frequency). Because signal power from respiration- and movement-based features can vary, values for these variables may need to be optimized more than once during scoring. In Spike2, choosing a small pixel window and a block size that includes the number of samples 2–4 times the sampling rate (in this case, a block size of 2048–4096 samples for a sampling rate of 1024 Hz) will achieve the best temporal and frequency resolution. It is important to note that spectrogram resolution also depends on the sampling rate of the data, and high sampling rates allow for better spectrogram and voltage trace resolution (Fig. 8B–D).

**Fig. 9:**
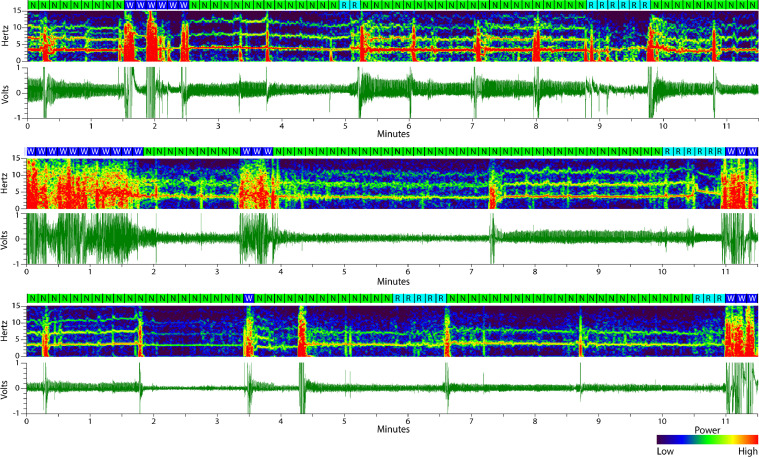
**Sleep visualized over minutes from data from different animals and recording days.** In conjunction with the supplemental files scored by experts, these examples show what respiration- and movement-based scoring looks like over several minutes and from separate animals and recording days. The boxes above each spectrogram indicate 10 s epochs with letters corresponding to each scored state: *W* = wake (blue), *N* = non-REM sleep (green), and *R* = REM sleep (cyan).

**Fig. 10:**
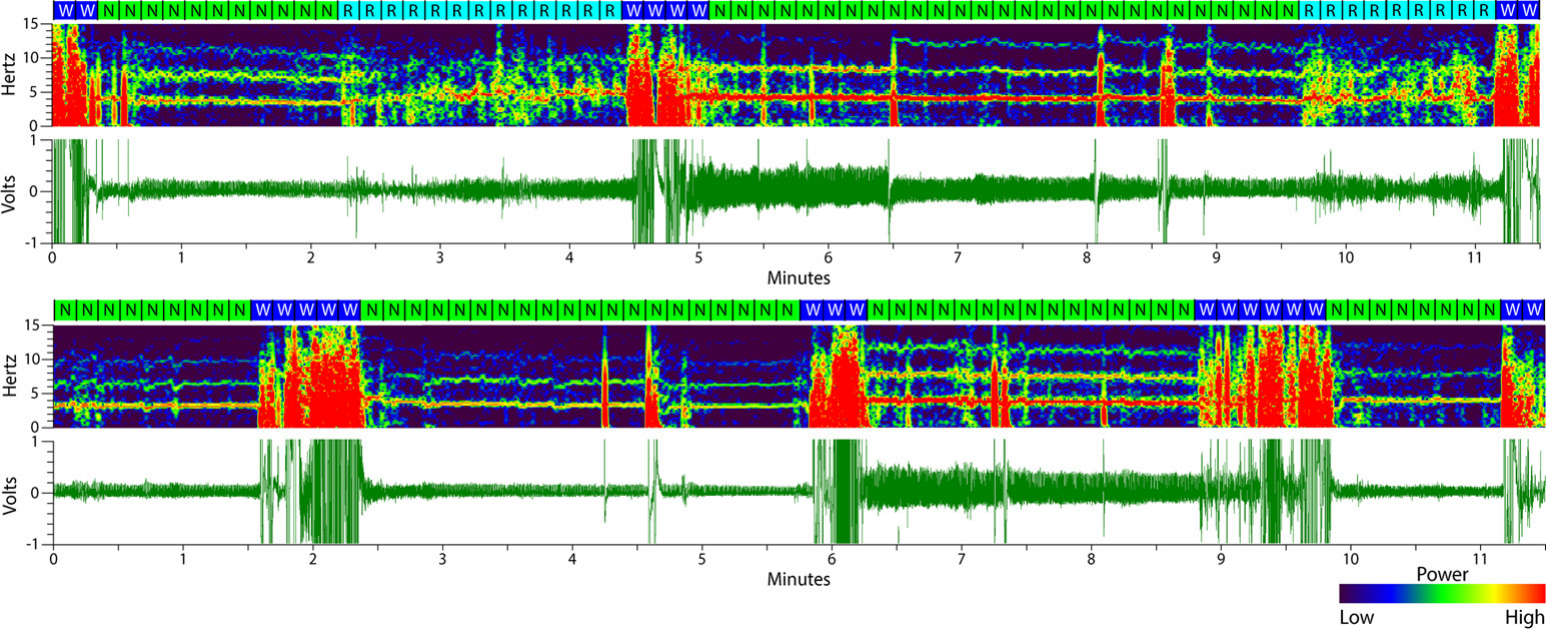
**Two additional sleep traces.** As in Fig. 9 and in conjunction with the supplemental files scored by experts, these examples show what respiration- and movement-based scoring looks like over several minutes and from separate animals and recording days. The boxes above each spectrogram indicate 10 s epochs with letters corresponding to each scored state: *W* = wake (blue), *N* = non-REM sleep (green), and *R* = REM sleep (cyan).

To assign arousal states to the data, an editable Spike2 “textmark” channel can be created using the “sleepscore_705″ script shared on the CED website (http://ced.co.uk/downloads/scriptspkanal). This script will enable the data to be subdivided into user-defined epoch lengths, assign manually-defined sleep states, plot a hypnogram, and export the data. Instructions for running the script are included with the script from CED. Briefly, once the data voltage trace and spectrogram have been created in Spike2, the script can be run using the “Script” menu. Click the “set-up” button at the menu at the top of the screen. A graphics user interface (GUI) will pop up in which you can select an epoch channel (the creation of the editable “textmark” channel), epoch duration, desired sleep states, time range, EEG banded spectrogram, and graphing options. The default sleep states are set for human sleep stages which subdivide non-REM sleep into multiple sub-states that are not used here for rodent sleep scoring. To change sleep state scoring options, check the “check to change stages” box to open a second GUI. Here, the appropriate arousal stage options (wake, non-REM sleep, and REM sleep) can be selected before clicking “ok” to close. Next, click on the “mark stages and events” button on the menu at the top of the screen. With this GUI open, select the desired state to score then hold the “control (ctrl)” button and drag the mouse across the epoch channel to assign the selected state. In this way, the data can be scored before being exported for further analysis and graphing (Figs. 9 and 10).

Supplemental file 1 contains the training document used to teach novices how to score sleep from respiratory and gross body movements collected by EF sensors. The instructions above are intended to enhance that training document by adding more detail, explanation, and scoring guidance.

## Declaration of Competing Interest

The authors declare the following financial interests/personal relationships which may be considered as potential competing interests:

HK and SH are co-inventors of US patent application 16/095,906, filed 10/23/2018, that includes use of EF sensor methodology for non-contact physio-behavioral monitoring of movements including respiration. NPP is a member of the scientific advisory board for Dixie Medical USA (unrelated to this work). LA has no disclosures.

## Appendix Supplementary materials

Supplemental files 3 and 5 are Spike2 filetype examples of data sleep-scored by experts. Supplemental files 2 and 4 are MATLAB filetype replicates of supplemental files 3 and 5. These files are intended for novices to hone their sleep scoring skills to match experts. Image, application 1

Image, application 2

Image, application 3

Image, application 4

Image, application 5
